# Supplementary material for: Controlled human infection with Plasmodium falciparum-infected mosquito bites elicits antibodies against mosquito salivary protein SG1L3
Source: PLoS Pathog. 2026 Oct 28;22(10):e1014719. doi: 10.1371/journal.ppat.1014719 (PMC13734598; doi:10.1371/journal.ppat.1014719)

Figure 1c western blot

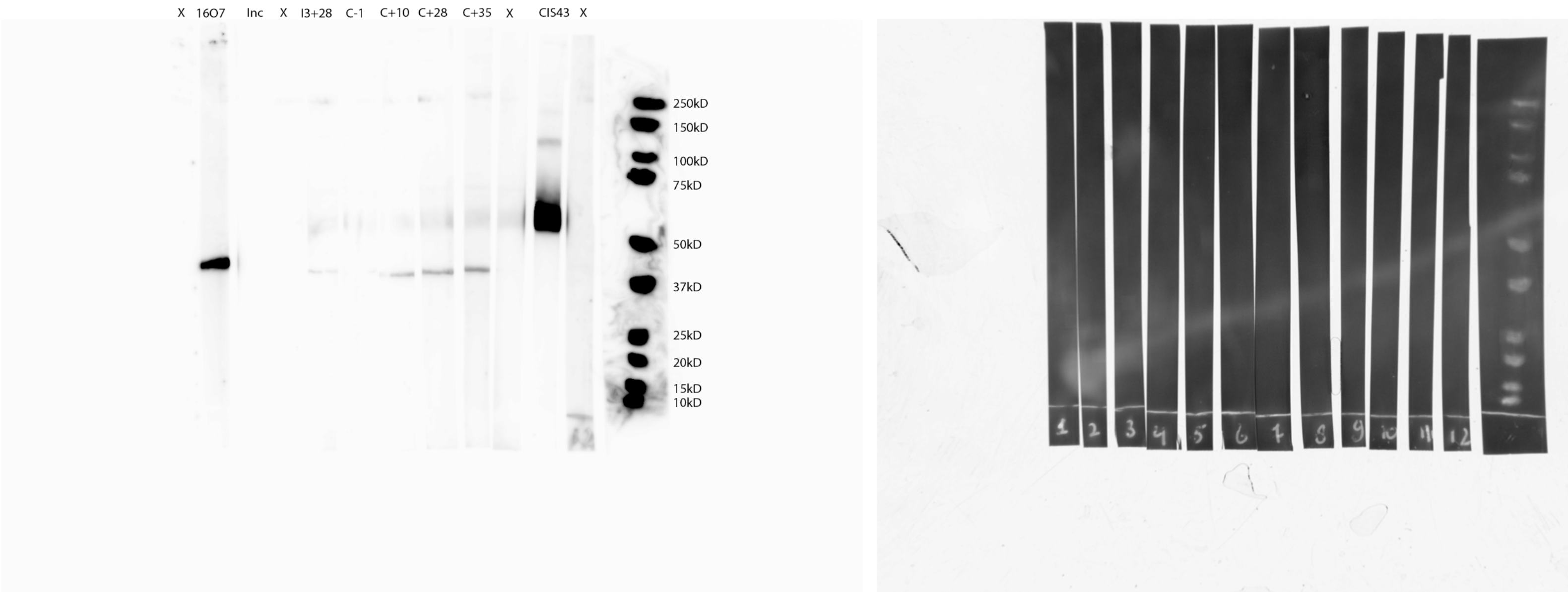

Figure 2a and 2c western blot (same blot, different contrast)

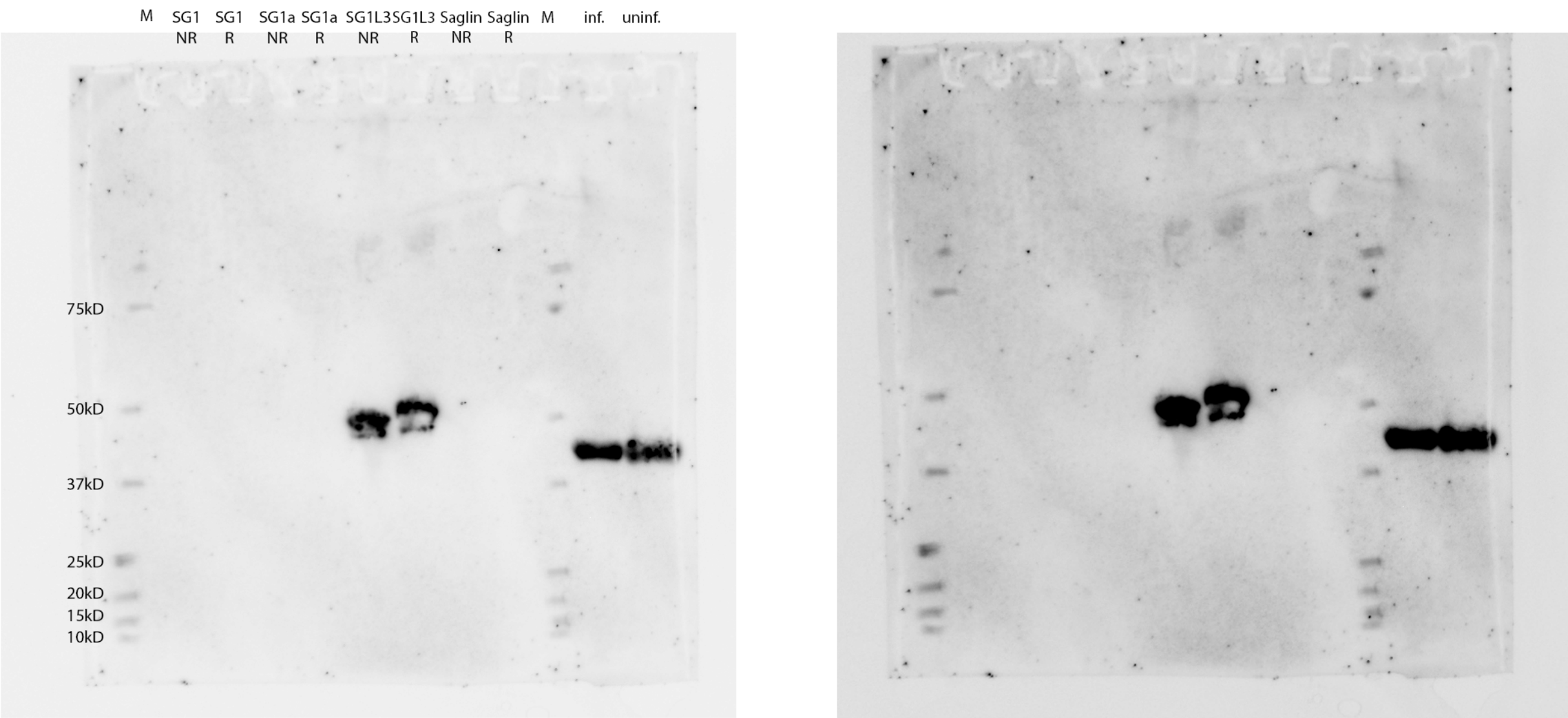

Sup Figure 2a, 2b and 2c western blot, coomassie and glycostain

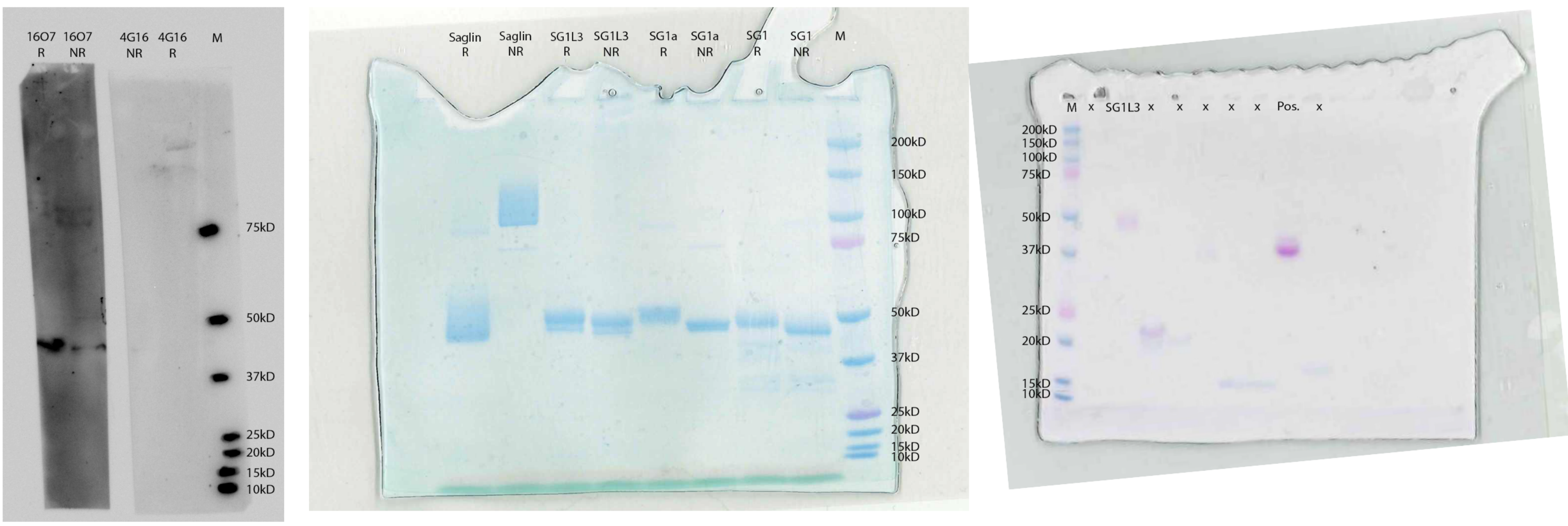

Supplementary figure 4b western blot (same blot, 2 or 30 sec exposure)

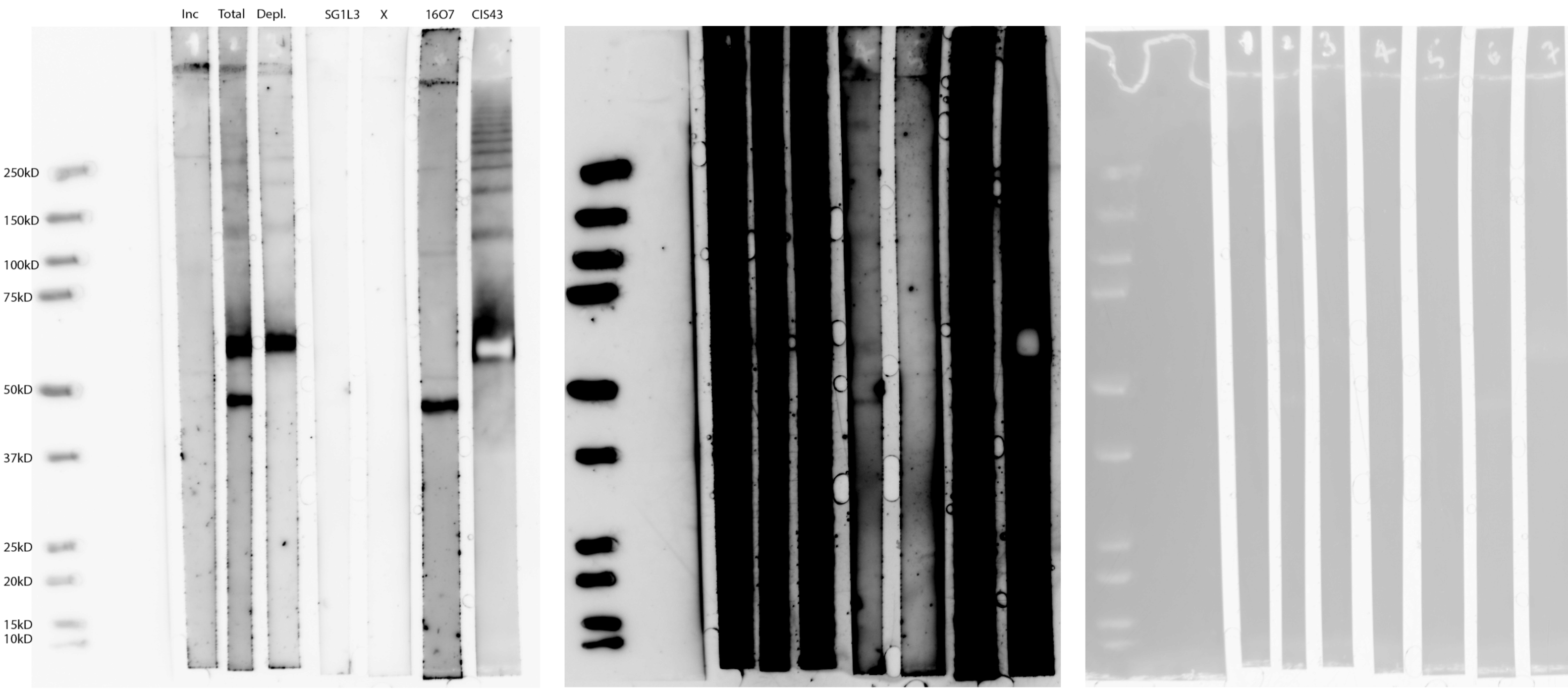

Supplementary figure 5c western blot

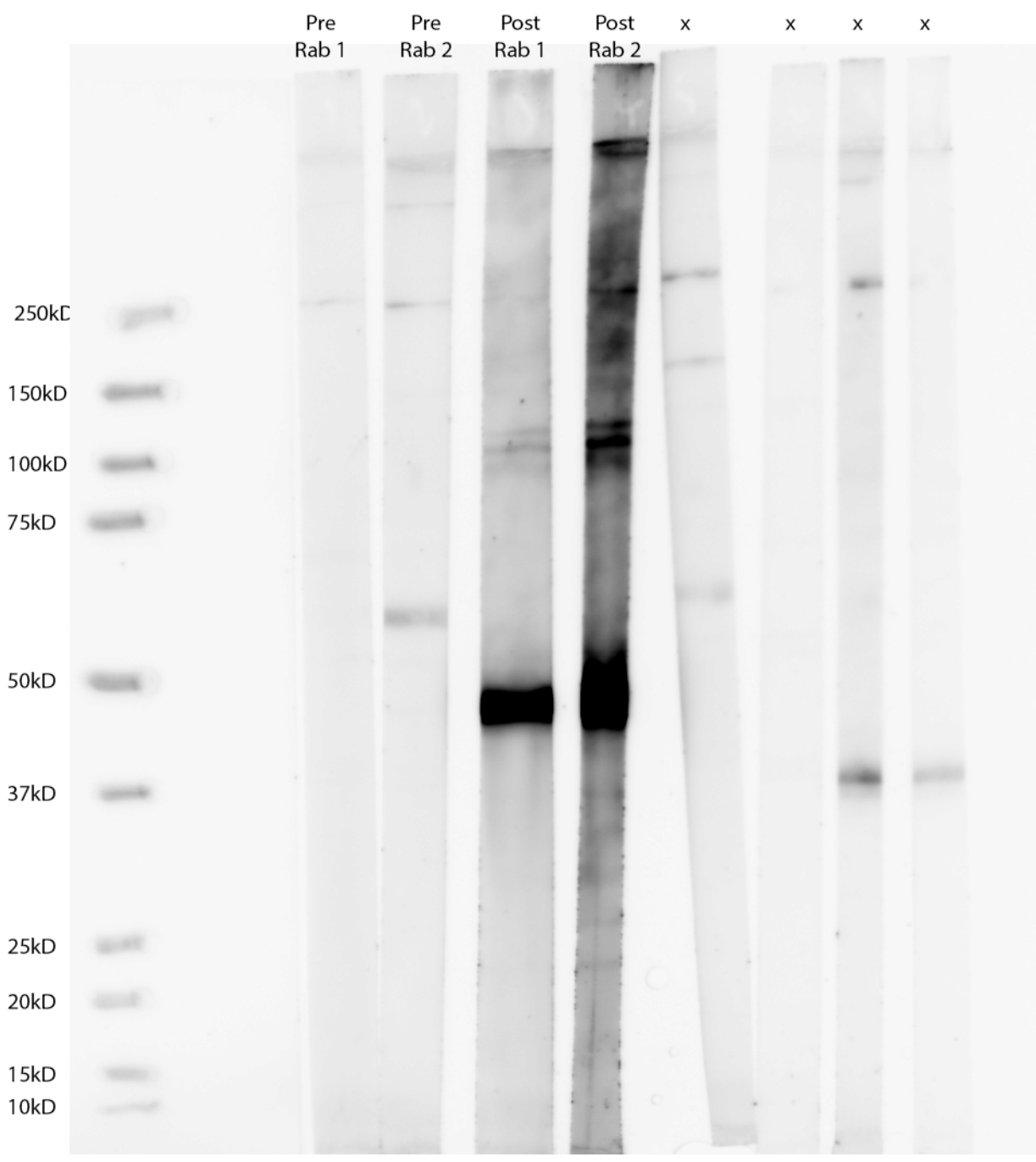

Supplement: Supplementary file 1 [file ppat.1014719.s008.pdf]
