# Supplementary material for: Controlled human infection with Plasmodium falciparum-infected mosquito bites elicits antibodies against mosquito salivary protein SG1L3
Source: PLoS Pathog. 2026 Oct 28;22(10):e1014719. doi: 10.1371/journal.ppat.1014719 (PMC13734598; doi:10.1371/journal.ppat.1014719)
Supplement: Supplementary file 2 [file ppat.1014719.s010.docx]

**Part I - Summary**

Reviewer #1: This paper by Andrade et al is the first to report on human antibodies elicited upon Plasmodium sporozoite vaccination that do not target the CSP, the major surface protein. This is very interesting, the work is well performed, and the paper is very well written. It should be noted that a previous paper doing about the same amount of work (reference 31) finding only CSP antibodies was published in Science.

Reviewer #2: This is well-executed work exploring samples from volunteers previously immunized with Plasmodium falciparum NF54 sporozoites through bites from infected Anopheles stephensi mosquitoes. The goal was to identify parasite targets, other than CSP, for antibodies that inhibit hepatocyte invasion by sporozoites.

The experiments are well-designed and surprisingly identified a protein from mosquito saliva, SG1L3, as the second major target for antibodies in the plasma of one volunteer, with CSP being the primary target.

Further well-designed experiments demonstrate that SG1L3 binds to the sporozoite surface, but antibodies against SG1L3 have no impact on sporozoite infectivity in vitro.

These are interesting and novel findings. The study's strengths include solid, well-designed experiments using unique samples from volunteers experimentally immunized through mosquito bites.

The weakness, however, is the lack of mechanistic insights. The authors should explore potential roles of SG1L3 binding to the sporozoite surface more thoroughly.

Reviewer #3: In this manuscript, Andrade et al., reported isolation of two distinct mAbs binding to mosquito salivary protein SG1L3 from controlled human infection volunteers. By thoroughly characterizing their binding and function, they report lack of sporozoite hepatocyte infection capacity of the mAbs. To confirm the functional role of targeting the protein SG1L3, they raised polyclonal antibodies against the protein in rabbits and showed similar lack of hepatocyte inhibition capacity in in vitro assays. Moreover, they showed increased frequency of antibody response against the protein SG1L3 as a factor of age and mosquito exposure in the sera samples collected from endemic residents from Burkina Faso. This manuscript report the first study performed on antibodies targeting the mosquito salivary gland protein and their functional consequence.

*We thank the reviewers for the positive evaluation of our work. Below we provide a detailed point-by-point reply to their concerns. We also include a revised manuscript with track changes based on the reviewer’s advice.*

**Part II – Major Issues: Key Experiments Required for Acceptance**

Reviewer #1: No major experiments needed, this is a very good and complete study.

*We thank the reviewer for their positive assessment of our manuscript.*

Reviewer #2: I think investigating how and why SG1L3 binds to the sporozoite surface is necessary.

How does it bind to sporozoites? The authors performed IP from uninfected mosquito salivary gland extracts. Would IP from infected mosquito salivary gland extracts provide information on potential receptors for SG1L3 on the sporozoite surface, or is the binding non-specific?

*Our binding assays showed that recombinant SG1L3 protein binds to sporozoites but not to gametes, suggesting that the binding of SG1L3 to the sporozoite surface is specific. Although we confirmed specific binding to sporozoites, we do not know the sporozoite receptor(s) involved in binding. We agree that immunoprecipitation and mass-spectrometry using our mAb(s) and infected salivary glands could provide more information on specificity and potential interaction partners for SG1L3 on the surface of the sporozoites. However, the current study focused on the identification of targets recognized by antibodies that were elicited following infectious mosquito bites and we therefore believe that identification of the SG1L3 receptor is out of scope. As part of a follow-up project, we are currently studying the role of SG1L3 in sporozoite infection in vivo, and hope to include identification and validation of the sporozoite receptor for SG1L3 there.*

Is it important for in vivo infections? The authors could perform mouse infections with P. berghei in the presence of anti-SG1L3 antibodies.

*In the current manuscript we were after non-CSP-specific antibodies (and their target) that were able to inhibit hepatocyte invasion in vitro and in a humanized mouse model (Fabra-Garcia et al 2022). Our experiments show that it is unlikely that SG1L3 plays a role in hepatocyte invasion. We agree with the reviewer that SG1L3 may indeed play a role during the skin-stage of sporozoite infection and suggested experiments to address this in the discussion section of our manuscript (lines 439-444). We are currently conducting a follow-up project that studies the role of SG1L3 in vivo; we strongly believe this work is out of the scope of the current manuscript.*

Is SG1L3 involved in salivary gland infection by sporozoites? SG1L3 can be silenced (PMID: 16530187). If not directly involved in SG infection (the most likely, considering the expression of the protein in the median lobe), are sporozoites without SG1L3 less infectious to hepatocytes or mice? There are many possible experiments to explore the potential roles of SG1L3 binding to sporozoites. Without this investigation, the overall significance of the manuscript is greatly reduced.

*As part of the follow-up project that we are currently running, we are setting up RNA interference experiments to indeed understand the role of SG1L3 protein in both human and mosquito infection. We politely disagree with the reviewer that without these experiments the significance of our current work is greatly reduced. In the current work, we report a novel methodology to screen B cells from immunized volunteers and were able to isolate mAbs to a target other than CSP, which has not been previously accomplished. Although we fully agree that the role of SG1L3 protein should be further investigated, we do believe it is beyond scope of the current publication.*

Reviewer #3: Line 170-171 and Fig 2f report high binding intensity of mAbs 16O7 and 4G16 to Pf sporozoites. What is the proportion of sporozoites that are coated by the mAbs? Does this proportion or MFI titrate as a function of mAb concentration? How do they compare to anti-PfCSP antibody (2A10) coating at the same concentration or titration series?Fig 3. Is 100 ug/ml of mAbs 16O7 and 4G16 used in hepatocyte invasion assay sufficient to coat ~100% of the Pf sporozoites? It would be good to clarify whether the lack of invasion inhibition is due to unbound sporozoites or lack of efficient coating.

*We previously observed, both by microscopy and flow cytometry, that the mAbs bind to the sporozoites but not to all sporozoites. To follow-up on this and address the reviewer’s comment, we have conducted a binding experiment in which we titrated mAbs 16O7 and 4G16 (SG1L3) and 2A10 (CSP) and calculated the percentage of sporozoites recognized by the mAbs. We found that SG1L3 is bound to approximately 30-40% of sporozoites It is indeed possible that the lack of invasion inhibition activity by mAbs 16O7 and 4G16 is due to the high amount (60-70%) of sporozoites that lack SG1L3 and are thus not recognized by the mAbs. This supports our conclusion that SG1L3 is unlikely to be the target of the functional non-CSP antibodies that we previously described in Fabra-Garcia et al.2022. We have now included the titration experiment in the supplementary fig 3 a, and have revised the text accordingly (lines 187-190). We have also included our interpretation and implications of these results in the discussion in the revised manuscript (lines 424-439).*

Have the authors considered the effect of anti-SG1L3 antibodies in combination with anti-PfCSP antibodies in sporozoite invasion inhibition? For example, any additive effects of anti-SGL1L3 mAbs can be determined by mixing the mAbs with sub-optimal concentration of 2A10 and measuring invasion inhibition. This will indicate potential aiding effects of targeting salivary gland proteins in sporozoite inhibition.

*We thank the reviewer for this suggestion and have tested if antibodies against SG1L3 can have a synergetic or additive effect on CSP-mediated inhibition. At a concentration of 2 µg/mL mAb 2A10 that conferred partial invasion inhibition, we showed that addition of 100 µg/ml of mixed mAbs 16O7 and 4G16 or 2 mg/ml rabbit purified IgGs did not enhance invasion inhibition. We have added these results to the revised manuscript (line 285-290 and figure 4 d).*

**Part III – Minor Issues: Editorial and Data Presentation Modifications**

Reviewer #1: Only minor cosmetic comments:
Please change reference 42 to the original paper published in Nature Medicine in 2006 by the same authors (the currently cited one is just a shorter version of the original one and doesn’t add to the literature).
*The reference has now been corrected to cite the original work by Amino et al.*

Figure 1: place panels A and B on top of each other and panel C to the right to not have so much white space in the figure

*We have now modified the layout of Figure 1 as suggested. We thank the reviewer for their assessment of our manuscript.*

Reviewer #2: The manuscript is clear. I have no minor issues.

*We thank the reviewer for their assessment of our manuscript.*

Reviewer #3: Line 125: From which time point of the clinical trial scheme, the B cells were isolated? Specifically the two mAbs were derived from B cells isolated early or late in the immunization scheme?
S1b: Which timepoint at which this inhibition was seen? Did it improve/boost over time within that individual? How does this timepoint correspond to the B cell isolation?

*We have now revised the manuscript (lines 126-128) to clearly state that the B cells were isolated from PBMCs collected 18 weeks post 3^rd^ immunization, one day before challenge (C-1 timepoint). We have also modified the corresponding supplementary figure legend to include this information. The functional sporozoite invasion inhibition assays in the previous work by Fabra-Garcia et al. 2022 were only performed with this time-point, so we do not know how functional activity developed following prime/boost vaccinations.*

Line 145: It would be good to show that 4G16 doesn’t recognize the same band in western.

*We have now included the western blot with SG1L3-containing sporozoite lysate that shows recognition by mAb 16O7 and no recognition by mAb 4G16, as supplementary figure 2a.*

If the antibodies prove non-functional in the proposed co-inhibition assay, I would recommend to include the non-inhibitory status in the title of the manuscript.

*At this point, we cannot rule out that SG1L3 plays a role during the skin stage of infection and that SG1L3-specific antibodies are active during this stage, as we describe in the discussion. We therefore prefer not to include non-functionality in the title. We believe the current title covers the work well and does not suggest that antibodies are active (or not).*

*We thank the reviewer for their assessment of our manuscript.*
