# Supplementary material for: Controlled human infection with Plasmodium falciparum-infected mosquito bites elicits antibodies against mosquito salivary protein SG1L3
Source: PLoS Pathog. 2026 Oct 28;22(10):e1014719. doi: 10.1371/journal.ppat.1014719 (PMC13734598; doi:10.1371/journal.ppat.1014719)
Supplement: Peer review history

# Peer review: Decision Letter 0

**DOI:** 10.1371/journal.ppat.1014719.r001

**Authors:** Marcelo Ferreira, Tracey Lamb

6 Jul 2026

PPATHOGENS-D-26-00985

Controlled human infection with Plasmodium falciparum-infected mosquito bites elicits antibodies against mosquito salivary protein SG1L3

PLOS Pathogens

Dear Dr. Jore,

Thank you for submitting your manuscript to PLOS Pathogens. After careful consideration, we feel that it has merit but does not fully meet PLOS Pathogens's publication criteria as it currently stands. Therefore, we invite you to submit a revised version of the manuscript that addresses the points raised during the review process.

Please submit your revised manuscript by Sep 04 2026 11:59PM. If you will need more time than this to complete your revisions, please reply to this message or contact the journal office at plospathogens@plos.org. When you're ready to submit your revision, log on to https://www.editorialmanager.com/ppathogens/ and select the 'Submissions Needing Revision' folder to locate your manuscript file.

Please include the following items when submitting your revised manuscript:

* A letter that responds to each point raised by the editor and reviewer(s). You should upload this letter as a separate file labeled 'Response to Reviewers'. This file does not need to include responses to any formatting updates and technical items listed in the 'Journal Requirements' section below.

* A marked-up copy of your manuscript that highlights changes made to the original version. You should upload this as a separate file labeled 'Revised Manuscript with Track Changes'.

* An unmarked version of your revised paper without tracked changes. You should upload this as a separate file labeled 'Manuscript'.

If you would like to make changes to your financial disclosure, competing interests statement, or data availability statement, please make these updates within the submission form at the time of resubmission. Guidelines for resubmitting your figure files are available below the reviewer comments at the end of this letter.

As the corresponding author, your ORCID iD is verified in the submission system and will appear in the published article. PLOS supports the use of ORCID, and we encourage all coauthors to register for an ORCID iD and use it as well. Please encourage your coauthors to verify their ORCID iD within the submission system before final acceptance, as unverified ORCID iDs will not appear in the published article. Only the individual author can complete the verification step; PLOS staff cannot verify ORCID iDs on behalf of authors.

We look forward to receiving your revised manuscript.

Kind regards,

Marcelo U. Ferreira, MD, PhD

Academic Editor

PLOS Pathogens

Tracey Lamb

Section Editor

PLOS Pathogens

Sumita Bhaduri-McIntosh

Editor-in-Chief

PLOS Pathogens

orcid.org/0000-0003-2946-9497

Michael Malim

Editor-in-Chief

PLOS Pathogens

orcid.org/0000-0002-7699-2064

**Additional Editor Comments (if provided):**

**Journal Requirements:**

If the reviewer comments include a recommendation to cite specific previously published works, please review and evaluate these publications to determine whether they are relevant and should be cited. There is no requirement to cite these works unless the editor has indicated otherwise.

1) Please ensure that the CRediT author contributions listed for every co-author are completed accurately and in full.

At this stage, the following Authors/Authors require contributions: Carolina M. Andrade, Renate C. van Daalen, Amanda Fabra-Garcia, Sanne Grievink, Geert-Jan van Gemert, Karina Teelen, Svenja Hester, Rianne Stoter, Marga van de Vegte-Bolmer, Chris Drakeley, Alfred B. Tiono, Robert W. Sauerwein, Teun Bousema, and Matthijs Jore. Please ensure that the full contributions of each author are acknowledged in the "Add/Edit/Remove Authors" section of our submission form.

The list of CRediT author contributions may be found here: https://journals.plos.org/plospathogens/s/authorship#loc-author-contributions

2) We ask that a manuscript source file is provided at Revision. Please upload your manuscript file as a .doc, .docx, .rtf or .tex. If you are providing a .tex file, please upload it under the item type u2018LaTeX Source Fileu2019 and leave your .pdf version as the item type u2018Manuscriptu2019.

3) We noticed that you used the phrase 'not shown' in the manuscript. We do not allow these references, as the PLOS data access policy requires that all data be either published with the manuscript or made available in a publicly accessible database. Please amend the supplementary material to include the referenced data or remove the references.

4) We do not publish any copyright or trademark symbols that usually accompany proprietary names, eg ©,  ®, or TM  (e.g. next to drug or reagent names). Therefore please remove all instances of trademark/copyright symbols throughout the text, including:

- ® on page: 17

- TM on pages: 11, 15, 17, 19, 20, and 21.

5) Please upload all main figures as separate Figure files in .tif or .eps format. For more information about how to convert and format your figure files please see our guidelines:

https://journals.plos.org/plospathogens/s/figures

6) Please upload a copy of Figure Fig 1-5 which you refer to in your text on pages 4, 5, 6, and 7. Or, if the figure is no longer to be included as part of the submission please remove all reference to it within the text.

7) We notice that your supplementary Figures, and Tables are included in the manuscript file. Please remove them and upload them with the file type 'Supporting Information'. Please ensure that each Supporting Information file has a legend listed in the manuscript after the references list.

8) We have amended your Competing Interest statement to comply with journal style. We kindly ask that you double check the statement and let us know if anything is incorrect.

9)PLOS now requires that authors provide the original uncropped and unadjusted images underlying all blot or gel results reported in a submission’s figures or Supporting Information files. This policy and the journal’s other requirements for blot/gel reporting and figure preparation are described in detail at https://journals.plos.org/plosone/s/figures#loc-blot-and-gel-reporting-requirements and https://journals.plos.org/plosone/s/figures#loc-preparing-figures-from-image-files.

When you submit your revised manuscript, please ensure that your figures adhere fully to these guidelines and provide the original underlying images for all blot or gel data reported in your submission. See the following link for instructions on providing the original image data: https://journals.plos.org/plosone/s/figures#loc-original-images-for-blots-and-gels.

In your cover letter, please note whether your blot/gel image data are in Supporting Information or posted at a public data repository, provide the repository URL if relevant, and provide specific details as to which raw blot/gel images, if any, are not available. Email us at customercare@plos.org if you have any questions.

**Reviewers' Comments:**

Reviewer's Responses to Questions

**Part I - Summary**

Please use this section to discuss strengths/weaknesses of study, novelty/significance, general execution and scholarship.

Reviewer #1: This paper by Andrade et al is the first to report on human antibodies elicited upon Plasmodium sporozoite vaccination that do not target the CSP, the major surface protein. This is very interesting, the work is well performed, and the paper is very well written. It should be noted that a previous paper doing about the same amount of work (reference 31) finding only CSP antibodies was published in Science.

Reviewer #2: This is well-executed work exploring samples from volunteers previously immunized with Plasmodium falciparum NF54 sporozoites through bites from infected Anopheles stephensi mosquitoes. The goal was to identify parasite targets, other than CSP, for antibodies that inhibit hepatocyte invasion by sporozoites.

The experiments are well-designed and surprisingly identified a protein from mosquito saliva, SG1L3, as the second major target for antibodies in the plasma of one volunteer, with CSP being the primary target.

Further well-designed experiments demonstrate that SG1L3 binds to the sporozoite surface, but antibodies against SG1L3 have no impact on sporozoite infectivity in vitro.

These are interesting and novel findings. The study's strengths include solid, well-designed experiments using unique samples from volunteers experimentally immunized through mosquito bites.

The weakness, however, is the lack of mechanistic insights. The authors should explore potential roles of SG1L3 binding to the sporozoite surface more thoroughly.

Reviewer #3: In this manuscript, Andrade et al., reported isolation of two distinct mAbs binding to mosquito salivary protein SG1L3 from controlled human infection volunteers. By thoroughly characterizing their binding and function, they report lack of sporozoite hepatocyte infection capacity of the mAbs. To confirm the functional role of targeting the protein SG1L3, they raised polyclonal antibodies against the protein in rabbits and showed similar lack of hepatocyte inhibition capacity in in vitro assays. Moreover, they showed increased frequency of antibody response against the protein SG1L3 as a factor of age and mosquito exposure in the sera samples collected from endemic residents from Burkina Faso. This manuscript report the first study performed on antibodies targeting the mosquito salivary gland protein and their functional consequence.

**********

**Part II – Major Issues: Key Experiments Required for Acceptance**

Please use this section to detail the key new experiments or modifications of existing experiments that should be absolutely required to validate study conclusions.

Generally, there should be no more than 3 such required experiments or major modifications for a "Major Revision" recommendation. If more than 3 experiments are necessary to validate the study conclusions, then you are encouraged to recommend "Reject".

Reviewer #1: No major experiments needed, this is a very good and complete study.

Reviewer #2: I think investigating how and why SG1L3 binds to the sporozoite surface is necessary.

How does it bind to sporozoites? The authors performed IP from uninfected mosquito salivary gland extracts. Would IP from infected mosquito salivary gland extracts provide information on potential receptors for SG1L3 on the sporozoite surface, or is the binding non-specific?

Is it important for in vivo infections? The authors could perform mouse infections with P. berghei in the presence of anti-SG1L3 antibodies.

Is SG1L3 involved in salivary gland infection by sporozoites? SG1L3 can be silenced (PMID: 16530187). If not directly involved in SG infection (the most likely, considering the expression of the protein in the median lobe), are sporozoites without SG1L3 less infectious to hepatocytes or mice?

There are many possible experiments to explore the potential roles of SG1L3 binding to sporozoites. Without this investigation, the overall significance of the manuscript is greatly reduced.

Reviewer #3: Line 170-171 and Fig 2f report high binding intensity of mAbs 16O7 and 4G16 to Pf sporozoites. What is the proportion of sporozoites that are coated by the mAbs? Does this proportion or MFI titrate as a function of mAb concentration? How do they compare to anti-PfCSP antibody (2A10) coating at the same concentration or titration series?

Fig 3. Is 100 ug/ml of mAbs 16O7 and 4G16 used in hepatocyte invasion assay sufficient to coat ~100% of the Pf sporozoites? It would be good to clarify whether the lack of invasion inhibition is due to unbound sporozoites or lack of efficient coating.

Have the authors considered the effect of anti-SG1L3 antibodies in combination with anti-PfCSP antibodies in sporozoite invasion inhibition? For example, any additive effects of anti-SGL1L3 mAbs can be determined by mixing the mAbs with sub-optimal concentration of 2A10 and measuring invasion inhibition. This will indicate potential aiding effects of targeting salivary gland proteins in sporozoite inhibition.

**********

**Part III – Minor Issues: Editorial and Data Presentation Modifications**

Please use this section for editorial suggestions as well as relatively minor modifications of existing data that would enhance clarity.

Reviewer #1: Only minor cosmetic comments:

Please change reference 42 to the original paper published in Nature Medicine in 2006 by the same authors (the currently cited one is just a shorter version of the original one and doesn’t add to the literature).

Figure 1: place panels A and B on top of each other and panel C to the right to not have so much white space in the figure

Reviewer #2: The manuscript is clear. I have no minor issues.

Reviewer #3: Line 125: From which time point of the clinical trial scheme, the B cells were isolated? Specifically the two mAbs were derived from B cells isolated early or late in the immunization scheme?

S1b: Which timepoint at which this inhibition was seen? Did it improve/boost over time within that individual? How does this timepoint correspond to the B cell isolation?

Line 145: It would be good to show that 4G16 doesn’t recognize the same band in western.

If the antibodies prove non-functional in the proposed co-inhibition assay, I would recommend to include the non-inhibitory status in the title of the manuscript.

**********

PLOS authors have the option to publish the peer review history of their article (what does this mean?). If published, this will include your full peer review and any attached files.

If you choose “no”, your identity will remain anonymous but your review may still be made public.

**Do you want your identity to be public for this peer review?**  For information about this choice, including consent withdrawal, please see our Privacy Policy.

Reviewer #1: No

Reviewer #2: No

Reviewer #3: **Yes:**  Rajagopal Murugan

[NOTE: If reviewer comments were submitted as an attachment file, they will be attached to this email and accessible via the submission site. Please log into your account, locate the manuscript record, and check for the action link "View Attachments". If this link does not appear, there are no attachment files.]

**Figure resubmission:**

**Reproducibility:**

To enhance the reproducibility of your results, we recommend that authors of applicable studies deposit laboratory protocols in protocols.io, where a protocol can be assigned its own identifier (DOI) such that it can be cited independently in the future. Additionally, PLOS ONE offers an option to publish peer-reviewed clinical study protocols. Read more information on sharing protocols at https://plos.org/protocols?utm_medium=editorial-email&utm_source=authorletters&utm_campaign=protocols

# Author comment: Author response to Decision Letter 1

**DOI:** 10.1371/journal.ppat.1014719.r002

12 Oct 2026

Attachment Submitted filename: Response to Reviewers.docx

# Editor report: Decision Letter 1

**DOI:** 10.1371/journal.ppat.1014719.r003

**Authors:** Marcelo Ferreira, Tracey Lamb

13 Oct 2026

Dear Dr. Jore,

We are pleased to inform that your manuscript, "Controlled human infection with Plasmodium falciparum-infected mosquito bites elicits antibodies against mosquito salivary protein SG1L3", has been editorially accepted for publication at PLOS Pathogens.

Before your manuscript can be formally accepted and sent to production, you will need to complete our formatting changes, which you will receive by email within a week. Please note that your manuscript will not be scheduled for publication until you have made the required changes.

IMPORTANT NOTES

(1) Copyediting and Proofreading: The corresponding author will receive a typeset proof for review, to ensure errors have not been introduced during production. Please review the PDF proof of your manuscript carefully, as this is the last chance to correct any errors. Please note that major changes, or those which affect the scientific understanding of the work, will likely cause delays to the publication date of your manuscript.

(2) Appropriate Figure Files: Please remove all name and figure # text from your figure files. Please also take this time to check that your figures are of high resolution, which will improve the readbility of your figures and help expedite your manuscript's publication. Please note that figures must have been originally created at 300dpi or higher. Do not manually increase the resolution of your files. For instructions on how to properly obtain high quality images, please review our Figure Guidelines, with examples at: http://journals.plos.org/plospathogens/s/figures.

We encourage you to use PACE (http://pace.apexcovantage.com/), a figure-checking tool which helps authors ensure all figures meet PLOS requirements so that figures can publish in the highest quality. PACE is a digital diagnostic and conversion tool for figure files. It will provide information about any failed check(s) and, if able, will automatically convert the figure into an acceptable file that passes quality checks.

To use PACE, you must first register as a user. Then, login and navigate to the UPLOAD tab, where you will find detailed instructions on how to use the tool.

(3) Striking Image: Please upload a striking still image to accompany your article if one is available (you can include a new image or an existing one from within your manuscript). Should your paper be accepted, this image will be considered for our monthly issue image and may also appear on our website to feature your article. Please upload this as a separate file, selecting "striking image" as the file type upon upload. Please also include a separate "Other" file with a caption, including credits and any potential copyright information. Please do not include the caption in the main article file. If your image is from someone other than yourself, please ensure that the artist has read and agreed to the terms and conditions of the Creative Commons Attribution License at http://journals.plos.org/plospathogens/s/content-license. Please note that PLOS cannot publish copyrighted images.

(4) Press Release or Related Media: If you or your institution will be preparing press materials for this manuscript, please inform our press team in advance at plospathogens@plos.org as soon as possible. We ask that you contact us within one week to plan ahead of our fast Production schedule. If you need to know your paper's publication date for related media purposes, you must coordinate with our press team, and your manuscript will remain under a strict press embargo until the publication date and time.

(5) Update your Profile Information: Now that your manuscript has been provisionally accepted, please log into Editorial Manager and update your profile, if needed. Go to http://www.editorialmanager.com/ppathogens, log in, and click on the "Update My Information" link at the top of the page. Please update your user information to ensure an efficient production and billing process.

(6) LaTeX users only: Our staff will ask you to upload a TEX file in addition to the PDF before the paper can be sent to typesetting, so please carefully review our Latex Guidelines http://journals.plos.org/plospathogens/s/latex in the meantime.

Thank you again for supporting Open Access publishing; we are looking forward to publishing your work in PLOS Pathogens.

Best regards,

Marcelo U. Ferreira

Academic Editor

PLOS Pathogens

Tracey Lamb

Section Editor

PLOS Pathogens

Dominique Soldati-Favre

Editor-in-Chief

PLOS Pathogens

***********************************************************

The authors have carefully revised the manuscript according to reviewers' suggestions.

# Editor report: Acceptance letter

**DOI:** 10.1371/journal.ppat.1014719.r004

**Authors:** Marcelo Ferreira, Tracey Lamb

Dear Dr. Jore,

We are delighted to inform you that your manuscript, "Controlled human infection with Plasmodium falciparum-infected mosquito bites elicits antibodies against mosquito salivary protein SG1L3," has been formally accepted for publication in PLOS Pathogens.

We have now passed your article onto the PLOS Production Department who will complete the rest of the pre-publication process. All authors will receive a confirmation email upon publication.

Thank you again for supporting open-access publishing; we are looking forward to publishing your work in PLOS Pathogens.

Best regards,

Dominique Soldati-Favre

Editor-in-Chief

PLOS Pathogens
